# COVID-19 outbreak after 100 days without community transmission: Epidemiological analysis of factors associated with death

**DOI:** 10.1016/j.heliyon.2023.e12941

**Published:** 2023-01-11

**Authors:** Nguyen Tran Minh Duc, Ali Ahmed-Fouad Abozaid, Le Van Truong, Nguyen Bao Hung, Dao Khanh Linh, Nguyen Hoang Dung, Teresa Pham Voong, Nguyen Tien Huy

**Affiliations:** a Faculty of Medicine, University of Medicine and Pharmacy at Ho Chi Minh City, Ho Chi Minh City, Viet Nam; b Faculty of Medicine, Ain Shams University, Cairo, Egypt; c Traditional Medicine Hospital, Ministry of the Public Security, Hanoi, Viet Nam; d Faculty of Public Health, University of Medicine and Pharmacy at Ho Chi Minh City, Ho Chi Minh City, Viet Nam; e Faculty of Medicine, Haiphong University of Medicine and Pharmacy, Hai Phong City, Viet Nam; f American University of Antigua, Osbourn, Antigua and Barbuda; g School of Tropical Medicine and Global Health, Nagasaki University, Nagasaki City, Japan

**Keywords:** Covid-19, SARS-CoV-2, Transmission, Death, Epidemiology, Outbreak, Nosocomial

## Abstract

**Background:**

Outbreak of SARS-CoV-2 pandemic has caused millions of deaths and lifelong consequences since December 2019. We attempted to evaluate the incidence, distribution, and risk factors associated with death after applying the social distance strategy to the second wave of SARS-CoV-2 in the Danang outbreak in Vietnam.

**Methods:**

We retrospectively reviewed the online the Danang Hospital reports, gathering the epidemiological history of confirmed SARS-CoV-2 patients. We then conducted a descriptive analysis of Fisher's Phi Coefficient and Cramer's, along with multiple logistic regression models to test the effects of symptomatology and control measures performed by Vietnamese government. The last report we examined on August 29, 2020.

**Results:**

A total of 389 SARS-CoV-2 confirmed cases were related to the Danang outbreak are included in our analysis with a mean age of 47.1 (SD = 18.4), involving 154 men and 235 women, with 34 cases of death and 355 were alive. The study showed significant results related to age, quarantine measures, previous negative SARS-CoV-2 test, and a range of symptoms, including shortness of breath and myalgia (p-value <0.05). Our multiple-variable analysis suggested the significant risk of death was related to age, severe symptomology, undetected SARS-CoV-2 test results, and prior quarantined SARS-CoV-2 history.

**Conclusions:**

Vietnamese authorities had implemented successful quarantine practices to control the SARS-CoV-2 outbreaks. However, this virus has shown dynamic spread beyond the ability of the country to control its transmission. Adequate screening, social distancing, and adequate care of elderly and healthcare workers can lower the risk of future outbreaks.

## 1 Introduction

The novel pandemic coronavirus (SARS-CoV-2, also known as COVID-19) disease, started in Wuhan City, China, in December 2019, has cumulatively increased to an estimated 140 million cases, according to the World Health Organization weekly report on April 18, 2021 [1]. Even after recovery, the SARS-CoV-2 could leave long-term pulmonary and cardiac complications [2]. This global crisis exceeds two previous Human Coronavirus pneumonia outbreaks, Severe Acute Respiratory Syndrome (SARS) in China in 2003 and Middle East Respiratory Syndrome (MERS), which started in Saudi Arabia in 2012. However, both previous virus strains did not adapt human to human transmission, which limited their spread, although their mortality rates were higher than the SARS-CoV-2 [3,4].

Despite having one of China's longest borders, the Vietnamese government showed a noteworthy successful control of the virus dispersal in the first wave on January 2020 and second wave on July 2020 that struck Vietnam. After 100 days without any community transmission on November 29, 2020, the numbers of cases recorded were 1341 to 35 deaths, most of which occurred during the second wave. The majority of them were related to the Danang Hospital outbreak that began on July 25, 2020 in Lien Chieu District, Danang City. The Danang authorities reported 368 total confirmed SARS-CoV-2 patients with 34 deaths based on data provided by the Danang government's official website [1,5,6].

At this time, several phase 1-2 clinical trials of SARS-CoV-2 vaccines had already been published. Governments around the world started vaccination campaigns giving hope for humanity in handling the widespread pandemic. We are not guaranteed the promise of a vaccine can eradicate the disease. Hence, the need for restrictive quarantine and surveillance measures including periodic screening of high-risk contacts, wearing face masks, cities partial or complete lockdown are necessary to avoid SARS-CoV-2 future outbreaks. Thus, our intention in this study is to evaluate the factors associated to death after quarantine practices and procedures, which were outlined by the Vietnamese Ministry of Health, on cases with symptomatic presentation of SARS-CoV-2 during the Danang outbreak [[7], [8], [9], [10]].

## 2 Methods

### 2.1 Data collection and study design

We have collected the information about SARS-CoV-2 in Danang patients retrospectively from the official website of public health system of Danang government website (https://opendata.danang.gov.vn) which included the reported infected cases in Danang City from the start of the outbreak in July 27, 2020 until August 29, 2020. The epicenter of this outbreak was the Danang Hospital, where the first case was discovered and isolated. Our scope was to evaluate the public health system and measures done by Vietnamese government in Danang City to contain the Covid-19 outbreak from further spread, besides estimating and discussing the factors related to death in this outbreak.

Initially, our study extracted data that included clinical symptomology incorporated with the main presentation of patients through the epidemiological history. At the time of diagnosis, the epidemiological investigation reviewed historical places, past contact details, and other personal information, especially age and gender. Ethics approval was not requested in our study since the data was already published online and had patients anonymized. The last report included in our data was published 29th August 2020 [5].

### 2.2 Level of contact tracing for COVID-19 in Vietnam

The Vietnamese government established a new plan to immediately control this outbreak and imposed a social distancing and large-scale covid-19 testing policies on 27th July. Investigations of identified exposure cases were categorized across 5 different levels based on exposure: F0 confirmed the nonidentifiable source of viral infection; Fn (n ranged 1–4), included those who directly contracted the infection through F (n-1) face to face interaction, talked to one another or spent some time together. They can be friends, domestic helpers, government officials who meet them directly, roommates, and restaurant servers as well. The quarantine measures applied to each level are illustrated in Fig. S1 [11].

### 2.3 Definition of the study variables and subgroups

All cases have been evaluated for clinical symptoms when diagnosed positive for Covid-19. There was no follow-up after the initial evaluation. The most common symptoms observed were: fever, cough, chills. Less common symptoms: myalgia, sore throat, diarrhea, red eyes, headache, loss of taste or smell, rash or redness, or cyanosis of the fingers or toes. Severe/critical symptoms included: requirement of intensive care unit (ICU) or mechanical ventilation, severe dyspnea up to inability of taking breath, persistent chest pain or pressure, hypoxia or hypoxemia, loss of consciousness, confusion, loss of ability to move or talk, or in severe cases cardiac arrest, severe arrhythmia, neural epilepsy or seizures [12].

The patient's past locations prior to quarantining have been considered through two categories of exposure tracing: the history of movement through 9 hospitals in Danang City (the Danang Hospital, the Lung Hospital, the Family Hospital, the 199 Hospital, the C Hospital, the Ung Buou Hospital, the Hoan My Hospital, the Dermatology Hospital, the Obstetrics and Gynecology Hospital), and history of moving to other locations in Danang City to the sub-district level. Information collected included personal contact information, age, gender, and previous COVID-19 reverse transcription polymerase chain reaction (RT-PCR) results. Data was subdivided according to death status, contact tracing level, and if the patient was symptomatic or asymptomatic, and entered into our descriptive data analysis.

### 2.4 Statistical analysis

From the extracted data, we evaluated patient characteristics related to mortality, contact, infection, and asymptomatic patient status by *t*-test, chi-square, phi coefficient, and Cramer's V. We use Stepwise Akaike information criterion (AIC) and Bayes methodology to define a model of patient classification according to optimal status through Modern Applied Statistics with S (MASS) and Bayesian Model Averaging (BMA) packages in the R language. After that, we conducted multivariate logistic regression to confirm the significance of our previous outcome of interest in a univariate model based on death prediction. All outcomes presented a mean (standard deviation), or Number of patients (%), and the cut-off p-value of significance was 0.05.

**Fig. 1:**
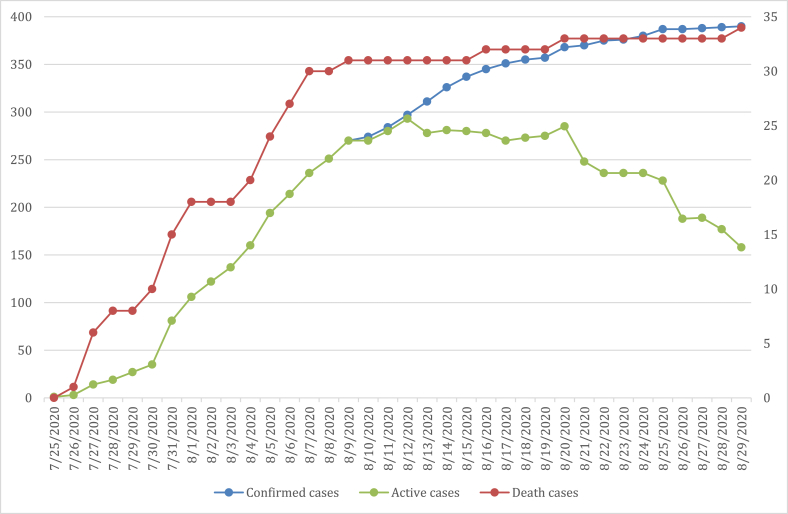
**Number of confirmed COVID-19 cases and death by date *(Danang outbreak, July 2020)*.** The red line (with the secondary y-axis) represents the number of Covid-19 infected patients who have died, indicating a rapidly increasing mortality in the period from 07/25/2020 to August 8, 2020. It also corresponds to the increase in active cases (the green line representing the y primary axis), and the blue line representing the cumulative number of confirmed cases also slowly increases at August 8, 2020 and until 8/29/2020 stopped completely at 389 cases. (For interpretation of the references to colour in this figure legend, the reader is referred to the Web version of this article.)

**Fig. 2:**
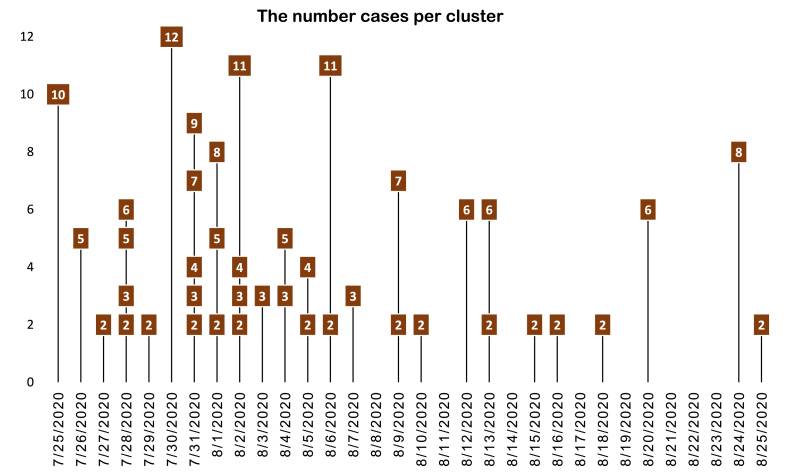
**Number of clusters identified each day during the outbreaks over time (with case F0) *(Danang outbreak, July 2020)*.** A total of 41 different clusters related to Danang COVID-19 outbreak represented by squares distributing by the date. The first case of each of these clusters had no known source of transmission (F0). The number inside these squares represent the number of cases per cluster. We identified 3 super spreader patients who infected ≥10 cases per cluster at days July 30, 2020, August 2, 2020 and August 6, 2020.

On the other hand, we used network analysis to identify clusters of infection through two factors: location and contact classification to build two different network models. According to the exposure investigation, model 1 evaluates the infection clusters and the locations moved through the 9 hospitals in Danang City. Model 2 is based on Model 1 with additional locations of subdistricts. The rising numbers of patients/clusters and the cumulative increase have been explained by Figs. 1 and 2.

## 3 Results

### 3.1 Number of included patients by date

A total of 389 confirmed COVID-19 Danang Hospital-associated cases were analyzed since its begin on July 25, until the last patient announced Aug 29 with mean age 47.1 (SD = 18.4), involving 154 men and 235 women (Table 1). The cumulative increase in the number of cases was fast growing to account for more than 290 cases in the first 15 days after imposing the social distancing and large screening testing measures, then gradually diminishing the next days of the outbreak until no new patients were recorded on Aug 29 (Fig. 1). Additionally, we identified approximately on the first fifteen days 29 different clusters of patients with their first case as F0 (non-identifiable source of infection). The number of each one reached up to 12 cases per cluster with three cluster more than 10 cases. After the quarantine actions applied by the Danang government, the number of confirmed cases decreased by half on subsequent days (Fig. 2). However, the number of deaths has risen dramatically through this outbreak to about 34 cases, of which thirty cases were reported only during the first two weeks (Fig. 1).

**Table 1:** Factors associated with mortality *(Danang outbreak, July 2020)*.

	Death	Survival	Total	p-value
	N = 34	N = 355	N = 389	
**Gender**				0.454
Male	16 (47.1%)	138 (38.9%)	154 (39.6%)	
Female	18 (52.9%)	217 (61.1%)	235 (60.4%)	
**Age (year)**	62.0 (14.6)	45.7 (18.2)	47.1 (18.4)	
				<0.001
**Five-degree contact tracing in Vietnam**^a^				0.011
F0	29 (85.3%)	191 (53.8%)	220 (56.6%)	
F1	3 (8.8%)	101 (28.5%)	104 (26.7%)	
F2	2 (5.9%)	38 (10.7%)	40 (10.3%)	
F3	0 (0.0%)	22 (6.2%)	22 (5.7%)	
F4	0 (0.0%)	3 (0.9%)	3 (0.8%)	
**Previously Covid-19 test**				0.001
Positive	1 (2.94%)	115 (32.4%)	116 (29.8%)	
Negative	33 (97.1%)	240 (67.6%)	273 (70.2%)	
**Quarantined before Covid-19 positive**				0.002
Yes	10 (29.4%)	206 (58.0%)	216 (55.5%)	
No	24 (70.6%)	149 (42.0%)	173 (44.5%)	
**Asymptomatic**				0.393
Yes	21 (61.8%)	250 (70.4%)	271 (69.7%)	
No	13 (38.2%)	105 (29.6%)	118 (30.3%)	
**Fever**				0.618
Yes	4 (11.8%)	53 (14.9%)	57 (14.7%)	
No	30 (88.2%)	302 (85.1%)	332 (85.3%)	
**Cough**				0.733
Yes	3 (8.82%)	38 (10.7%)	41 (10.5%)	
No	31 (91.2%)	317 (89.3%)	348 (89.5%)	
**Shortness of breath**				<0.001
Yes	6 (17.6%)	8 (2.3%)	14 (3.6%)	
No	28 (82.4%)	347 (97.7%)	375 (96.4%)	
**Nasal congestion/runny nose**				0.130
Yes	1 (2.9%)	2 (0.6%)	3 (0.8%)	
No	33 (97.1%)	353 (99.4%)	386 (99.2%)	
**Myalgia**				0.038
Yes	1 (2.9%)	1 (0.3%)	2 (0.51%)	
No	33 (97.1%)	354 (99.7%)	387 (99.5%)	
**Chills**				0.022
Yes	8 (23.5%)	37 (10.4%)	45 (11.6%)	
No	26 (76.5%)	318 (89.6%)	344 (88.4%)	
**Sore throat**				0.413
Yes	1 (2.9%)	23 (6.5%)	24 (6.2%)	
No	33 (97.1%)	332 (93.5%)	365 (93.8%)	
**Headache**				0.222
Yes	0 (0.0%)	15 (4.2%)	15 (3.9%)	
No	34 (100%)	340 (95.8%)	374 (96.1%)	
**Taste/smell**				0.591
Yes	0 (0.00%)	3 (0.9%)	3 (0.8%)	
No	34 (100%)	352 (99.2%)	386 (99.2%)	
**Chest pain**				0.799
Yes	1 (2.9%)	8 (2.3%)	9 (2.3%)	
No	33 (97.1%)	347 (97.7%)	380 (97.7%)	
**Total symptoms**				0.013
0	21 (61.8%)	250 (70.4%)	271 (69.7%)	
1	5 (14.7%)	47 (13.2%)	52 (13.4%)	
2	6 (17.6%)	35 (9.9%)	41 (10.5%)	
3	0 (0.0%)	21 (5.9%)	21 (5.4%)	
4	2 (5.9%)	2 (0.6%)	4 (1.0%)	
**Common symptoms**^b^				0.696
0	23 (67.6%)	263 (74.1%)	286 (73.5%)	
1	8 (23.5%)	61 (17.2%)	69 (17.7%)	
2	2 (5.9%)	26 (7.3%)	28 (7.2%)	
3	1 (2.9%)	5 (1.4%)	6 (1.5%)	
**Less common symptoms**^c^				0.638
0	32 (94.1%)	318 (89.6%)	350 (90.0%)	
1	2 (5.9%)	32 (9.0%)	34 (8.7%)	
2	0 (0.0%)	5 (1.4%)	5 (1.3%)	
**Severe symptoms**^d^				0.001
0	27 (79.4%)	341 (96.1%)	368 (94.6%)	
1	7 (20.6%)	12 (3.4%)	19 (4.9%)	
2	0 (0.00%)	2 (0.6%)	2 (0.5%)	

Statistical analysis test: a: Fisher's exact test, b: Phi and Cramer's V.

Descriptive information was reported as a: N (%), b: mean (SD).

a F0 was non-identifiable source COVID-19 infected patient, Fn the one who contracted the infection from Fn-1.

b Common symptoms: fever, cough, chills.

c Less common symptoms: myalgia, sore throat, diarrhea, red eyes, headache, loss of taste or smell, rash or redness, or cyanosis of the fingers or toes.

d Severe symptoms: needing the intensive care unit (ICU) or mechanical ventilation, severe dyspnea up to inability of taking breath, persistent chest pain or pressure, hypoxia or hypoxemia up to the loss of consciousness level of confusion, loss of ability to move or talk in addition to cardiac arrest, severe arrhythmia, neural epilepsy or seizures.

### 3.2 Classification of included patients by place

**Fig. 3:**
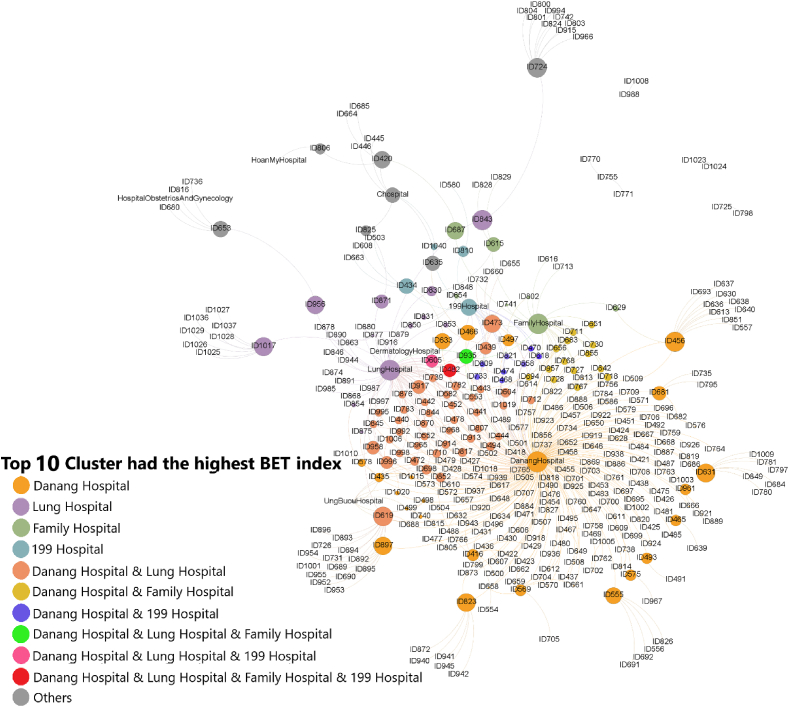
**Network between COVID-19 cases and hospitals in Danang City *(Danang outbreak, July 2020)*.** The map showed the relationships between the patients and their indirect relationships through the hospitals represented by a graph contraction. The size of these circles is increased according to the number of associations. We mark the top 3 clusters with the highest infection rates, the Danang Hospital (in orange), the Lung Hospital cluster (in purple) the Family Hospital (in cyan) and the 199 Hospital (in grey blue). The BET index is used to evaluate the frequency of links between two random nodes in any lattice. The node has a higher BET level, the higher the influence in the network. In our chart, the Danang Hospital's BET is the highest figure as the reference value, the second is the Lung Hospital 's BET of 0.28 and the third is the Family Hospital's BET of 0.07, which reflects these are the two centers linking COVID-19 cases in Danang. This could imply that these two outbreaks are directly related and are the main drivers of the outbreak in Danang We provided an online interactive version on this link: https://alifouad98.github.io/Figure-3/. (For interpretation of the references to colour in this figure legend, the reader is referred to the Web version of this article.)

In our drawing network, most of the Danang outbreak cases were linked directly to the Danang Hospital, the Lung Hospital, the Family Hospital and the 199 Hospital (Fig. 3). Furthermore, the data displayed the super spread factor (patients) connected to more than one place/hospital, such as ID456, ID619, and ID724. The highest number of mortality was found in hospitalized cases prior to COVID-19 screening test having an unknown infection source (F0) shown in Table S1.

### 3.3 Descriptive outcomes of patients

Our descriptive analysis determined significant values in many factors that indicated their prevalence in mortality. These factors were significant only in the previous negative tests for COVID-19, previously quarantined patients (97.1% and 70.6%, p-value = 0.001 and 0.002 respectively) and for contact tracing Vietnamese measures (p-value = 0.011) but highly significant concerning age component (mean = 62, SD = 14.6, **p-value** < **0.001**). Regarding the patients’ main presentations, we found remarkably that severe symptomatology was largely involved in nonsurvivors with a p-value equal to 0.001. Besides, we noticed them broadly affected with shortness of breath, myalgia, and chills (p-value = <0.001, 0.038, and 0.022, respectively) (Table 1).

Table 2 showed the comparison between the symptomatic and asymptomatic patients, which demonstrated statistical significance attributed to symptomatic patients in quarantine (p-value = 0.001) and level of contact history (p-value = 0.035). Alternatively, we discovered in Table S2 the significance was more prominent in age (mean = 50.1, SD = 17.2, p-value = 0.004). Patients with existing chest pain, previous negative Covid-19 results, and quarantined before Covid-19 positive history were at significantly higher risk in the F0 group compared to F1-4 (p-value = 0.004, 0.003, and 0.014 respectively) (Table S2).

**Table 2:** Factors associated with asymptomatic (Danang outbreak, July 2020).

	Symptomatic	Asymptomatic	p-value
	N = 118	N = 271	
**Gender**			0.961
Male	46 (39.0%)	108 (39.9%)	
Female	72 (61.0%)	163 (60.1%)	
**Age (year)**	45.7 (18.6)	47.7 (18.4)	0.344
**Five-level of contact tracing in Vietnam**^a^			0.035
F0	64 (54.2%)	156 (57.6%)	
F1	41 (34.7%)	63 (23.2%)	
F2	10 (8.5%)	30 (11.1%)	
F3	2 (1.7%)	20 (7.4%)	
F4	1 (0.85%)	2 (0.7%)	
**Previously COVID-19 test**			0.374
Negative	31 (26.3%)	85 (31.4%)	
Positive	87 (73.7%)	186 (68.6%)	
**Quarantined before COVID19 positive**			0.001
Yes	50 (42.4%)	166 (61.3%)	
No	68 (57.6%)	105 (38.7%)	

Statistical analysis test: a: Fisher's exact test, b: Phi and Cramer's V.

Descriptive information was reported as a: N (%), b: mean (SD).

a F0 was non-identifiable source COVID-19 infected patient, Fn the one who contracted the infection from Fn-1.

The prevalence of outcomes according to the source of infection with a mortality rate that was higher for nonidentifiable sources (F0) (85.3%, n = 29, total = 220) than the identifiable one (F1–F4) (14.7%, n = 5, total = 169) as shown in Table 1. Mortality was seen in patients with more severe symptoms (6.4% and 3.8%, n = 14 and 7), but the least total symptoms (29.1% and 31.95%, n = 64 and 54) and common symptoms (25.9% and 27.2, n = 57 and 46) respectively (Table S2). The majority of F0 patients were asymptomatic (57.6%, n = 156) when they compared to (F1–F4) ones (42.4%, n = 115) (Table 2).

### 3.4 Uni- and multi-variate analysis for specific outcomes

Our uni- and multivariate analysis proved the relative susceptibility of death in patients with previously negative test, quarantined before COVID-19 was positive, and severe symptoms (OR = 0.06, 0.32 and 5.09) [95%CI: 0–0.29, 0.13–0.72 and 1.45–17.66, p-value = 0.006, 0.008 and 0.01] respectively. The data demonstrated a high correlation with the age covariate (OR = 1.78) [95%CI: 1.39–2.36, **p-value** < **0.001**]. Although, the defined sources of infection (F1-4) resulted significantly in our univariate analysis (OR = 0.2) [95%CI: 0.1–0.22, p-value = 0.001]. The multivariable model did not show any association (OR = 0.38) [95%CI: 0.12–1.01, p-value = 0.069] (Table S3).

## 4 Discussion

### 4.1 What is already known on the topic and what this study adds

The Danang local coronavirus outbreak was considered the most extensive outbreak in Vietnam after 3 months without community cases. Our data evaluated 389 COVID-19 cases in this period with an estimated 8.7% mortality rate, which is mostly associated with older age, delayed detection (undetected COVID-19 test results), positive after quarantine, and presented with severe symptoms at the time of diagnosis.

The rapid increase in the infection rate of the easily transmitted virus COVID-19 has stricken the world from its beginning in Dec 2019 until the present day, indicating the necessity of countries to strict measures to put an end to this pandemic. The second wave of SARS-COV-2 can be worse than the first one due to the rapid mutation seen in this virus, showing easier dynamic transmissibility. There are various factors, including airborne transmission of aerosols through undetermined causes such as direct expiratory actions (coughing and/or sneezing) or indirect contact with contaminated sources from infected carriers. Asadi et al. [13], discussed, that most infections occurred during the asymptomatic or pre-asymptomatic phase, which aided by the rapid dissemination of SARS-CoV-2. In other words, the infection shows a dynamic transmission beyond the main airborne or respiratory mechanisms (coughing or sneezing). However, this study has shown that the virus can be suppressed by social distancing. Our study substantially proved in several ways, most of the confirmed patients were asymptomatic at the time of diagnosis (69.7%, n = 271), whom 57.6% of them were undefined sources (n = 156). After implementing the social distance policy, the infection rate started to decrease rapidly after 15 days indicating the success of measures taken by Vietnamese government, and establishing that approximately 95% of patients have a maximum incubation period of 15 days. The effectiveness of the social distancing and quarantine strategies was shown after 15 days of implementation. (Tables 1 and 2 and Fig. 1) [13,14].

The question here is whether or not we can recognize asymptomatic patients before developing COVID-19 symptoms. Clinically, there is no association in the risk of death attributed to the asymptomatic patients in our study (Table 1). Theoretically, the development of contact tracing and early detection of COVID-19 patients can help in the containment of current and future outbreaks. Vietnam developed an excellent contract tracing system that can detect early symptomatic cases. Moghadas et al. study proved that these actions could reduce the risk of future outbreaks below 1% by isolating the identified exposure cases early [15].

Subsequently, successful measures can be implemented to isolate asymptomatic cases. The most important was through direct mass screening of COVID using RT-PCR test or computed tomography (CT) examination for all contacts of prespecified COVID-19 patients. Additional measures implemented were contacted tracing for all visitors to locations identified as a source of outbreaks and those who recently came from the outbreak epicenters, social distancing, quarantine, and lockdown policies for cities that are new epicenters of COVID-19 [[16], [17], [18], [19], [20], [21]]. Interestingly, we have discovered that a previous negative RT-PCR result can suggest higher mortality risk (p-value = 0.006). The higher mortality rate could result from exposure of negative RT-PCR patients at screening locations at the same time as potentially positive RT-PCR patients. After a negative test, patients can still develop severe symptoms during the prodromal period of the virus. A study in Brazil showed a high mortality rate is linked to delayed diagnosis and lower social-economic individuals [22]. need for repeated RT-PCR tests at least 2 times within an interval of 3 days in between tests as recommended by Ai et al. [23], who suggested a combination of CT scan and examination can detect around 70% of previous negative RT-PCR patients. Another factor we discovered in this study, prior quarantine patients before a positive COVID-19 test, could raise the mortality rate of individuals in this group (p-value = 0.002) (Table 1). It might be possible that quarantined patients were not monitored daily by healthcare providers if they acquired any symptoms instead of depending on self-reporting of symptoms from quarantine patients. Late reporting of symptoms by quarantined patients delayed the confirmed diagnosis and initiation of treatment, therefore resulting in a higher mortality risk [23].

In general, the risk of death could be defined by several elements. The age component was highly associated with noting that increased age was more likely associated with a higher mortality rate (**p-value** < **0.001**) due to the increasing comorbidity among older populations. An existing study showed the most common comorbidities among the geriatric population are hypertension (48.8%) and chronic obstructive pulmonary disease (COPD) (29%). In another study, we found that the case fatality of COVID-19 can increase from 8% up to 14% for those ≥70 years old [24,25]. Furthermore, severe symptomatology could play a role as well for mortality risk. We estimated the p-value of such cases equal to 0.01, which is consistent with many published studies’ outcomes. Zhang et al. found around 83% of mortality cases entered the ICU, 80.5% of these cases were older than 60, and about 69.5% related to respiratory failure as the leading cause of death, which is relatively similar to our result of the shortness of breath category (**p-value** < **0.001**) presented in Table 1 [26].

Super-spreader factors (patients) existed in the Danang outbreak (Table S1 and Fig. 3). In the beginning, super-spreaders were **mostly** asymptomatic or mildly symptomatic patients who actively traveled to various crowded places/non-identified sources (**F0**) and infected ≥10 cases. An example of such a case occurred in Korea, in which a female patient transmitted the infection to more than 5000 individuals at Shincheonji Church of Jesus in Daegu. This event was recorded as the first large outbreak outside of China [27,28]. case in Ningbo City in China transmitted COVID-19 to 28 cases [29]. In our study, we have three F0 patients who infected ≥10 sequence (ID456, ID619, and ID724).

On the other hand, we have noticed that most mortality cases were F0 and showed high correlation in our univariate analysis (p-value = 0.001) (Table S3), despite the nonsignificance of the multivariate result. We hypothesized that the spread of the virus was associated with hospitalized status and increased liability to provoke severe symptoms. The diagnosis was made very late, unlike most of the contacts (F1-4) discovered and monitored early. That brings us to the conclusion that the most dangerous outbreaks were those associated with local hospitals. In Vietnam, more than 97% of cases of death were connected to the Danang Hospital. The role of healthcare workers in spreading the infections seems to be highly related. Hence, the country needs to provide protective tools, equipment, and appropriate infection control measures. Gan et al. recommended the Singaporean Ministry of Health strategy to manage all hospital-infected cases within the same hospital, plus initializing the contact tracing system, which was similar to the Vietnamese strategy [30]. However, we must protect our healthcare personnel from exposure and risk of contracting Covid-19 since healthcare personnel are responsible for caring for multiple patients throughout the day. They could become a “super spreader” without any symptoms to a vulnerable population of hospitalized patients and the community in which they live and serve. By implementing a regularly screening protocols of healthcare personnel, especially those in direct contact with Covid-19 patients regardless of the presentation of Covid-19 symptoms. In addition to wearing face masks or shields, we need to prepare public hospitals with high-quality ICU types of equipment for severe/critical cases of SARS-CoV-2 pneumonia.

### 4.2 Limitations of this study

Our study, based on the findings of epidemiological investigations, is considered to be an effective method for assisting Vietnam in effectively quarantining and controlling COVID-19 in Danang City. There are, however, certain restrictions. The findings' generalizability is questioned due to the small sample sizes of reported cases and the majority of patient data coming from a small number of hospitals. The study's unfeasible characteristics transpired due to the unknown source of transmission due to the lack of performance genetic analysis, and the patient's symptoms were only followed up on when the patient was confirmed positive for COVID-19.

## 5 Conclusion

Vietnamese authorities implemented appropriate quarantine practices and procedures to control the COVID-19 outbreaks seen in Danang. However, this virus has shown a dynamic shift beyond the ability of countries to control COVID-19 transmission. Controlling asymptomatic or pre-symptomatic super-spreader cases is crucial to maintaining the lower risk of outbreak. Supporting hospitals and preventing cross-infection through infection-controlled policies, especially for the elderly population and health-care workers, are vital in containing the spread of the virus.

## Author contribution statement

Nguyen Tran Minh Duc: Conceived and designed the experiments; Performed the experiments; Analyzed and interpreted the data; Wrote the paper. Ali Ahmed-Fouad Abozaid: Performed the experiments; Analyzed and interpreted the data; Contributed reagents, materials, analysis tools or data; Wrote the paper. Le Van Truong, Teresa Pham Voong: Contributed reagents, materials, analysis tools or data; Wrote the paper. Nguyen Bao Hung, Dao Khanh Linh, Nguyen Hoang Dung: Performed the experiments; Analyzed and interpreted the data. Nguyen Tien Huy: Conceived and designed the experiments; Wrote the paper.

## Funding statement

This research did not receive any specific grant from funding agencies in the public, commercial, or not-for-profit sectors.

## Data availability statement

Data will be made available on request.

## Declaration of competing interest

The authors declare no competing interests.
