# Supplementary material for: COVID-19 outbreak after 100 days without community transmission: Epidemiological analysis of factors associated with death
Source: Heliyon. 2023 Jan 11;9(1):e12941. doi: 10.1016/j.heliyon.2023.e12941 (PMC9832690; doi:10.1016/j.heliyon.2023.e12941)
Supplement: Multimedia component 1 [file mmc1.docx]

**Supporting information:**

**Fig. S1: Classification of exposures to patients infected with Covid-19.** this diagram showed the five-level Vietnamese government contact tracing plan which was applied on Danang outbreak.

**Table S1: Dataset of 34 Covid-19 death cases in Danang.**

**Table S2: Summary descriptive table by groups of level of contact tracing**

**Table S3: Summary of Uni- and multivariable logistic regression analysis for death patients**


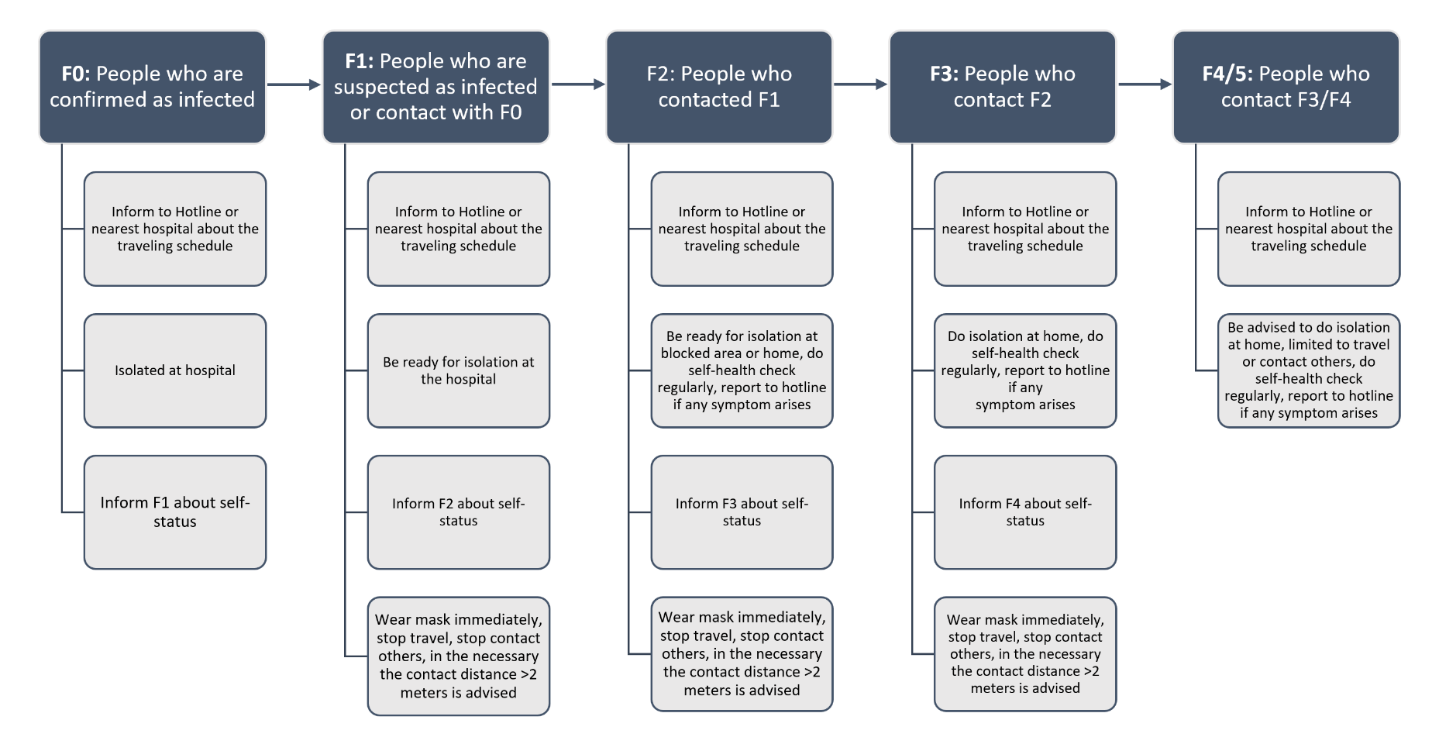


**Fig. S1: Classification of exposures to patients infected with Covid-19**

**Table S1: Dataset of 34 COVID-19 death cases in Danang *(Danang outbreak, July 2020)***

| ID | Date | Cluster | Type cases* | Symptoms | Hospitals |
| --- | --- | --- | --- | --- | --- |
| ID418 | 7/26/2020 | ID418 | F0 | Fever; Cough; Shortness of breath; Chills | Danang Hospital |
| ID426 | 7/27/2020 | ID426 | F0 | Shortness of breath; Chills | Danang Hospital |
|  |  |  |  |  |  |
| ID428 | 7/27/2020 | ID428 | F0 | Chills; Chest pain | Danang Hospital |
| ID429 | 7/27/2020 | ID416 | F2 | Cough; Myalgia | Danang Hospital |
| ID430 | 7/27/2020 | ID416 | F2 | Asymptomatic | Danang Hospital |
| ID431 | 7/27/2020 | ID416 | F1 | Shortness of breath; Chills | Danang Hospital |
| ID436 | 7/28/2020 | ID436 | F0 | Nasal congestion/Runny nose | Danang Hospital |
| ID437 | 7/28/2020 | ID437 | F0 | Shortness of breath; Chills | Danang Hospital |
| ID453 | 7/30/2020 | ID453 | F0 | Asymptomatic | Danang Hospital |
| ID456 | 7/30/2020 | ID456 | F0 | Fever; Cough | Danang Hospital |
| ID475 | 7/31/2020 | ID475 | F0 | Asymptomatic | Danang Hospital |
| ID479 | 7/31/2020 | ID479 | F0 | Asymptomatic | Danang Hospital |
| ID485 | 7/31/2020 | ID485 | F0 | Chills | Danang Hospital |
| ID496 | 7/31/2020 | ID496 | F0 | Asymptomatic | Danang Hospital |
| ID499 | 7/31/2020 | ID498 | F1 | Fever | Danang Hospital; Ung Buou Hospital |
| ID575 | 8/1/2020 | ID575 | F0 | Asymptomatic | Danang Hospital |
| ID577 | 8/1/2020 | ID577 | F0 | Chills | Danang Hospital |
| ID585 | 8/1/2020 | ID585 | F0 | Asymptomatic | None |
| ID651 | 8/4/2020 | ID651 | F0 | Asymptomatic | Danang Hospital |
| ID666 | 8/4/2020 | ID666 | F0 | Asymptomatic | Danang Hospital |
| ID696 | 8/5/2020 | ID696 | F0 | Asymptomatic | Danang Hospital; Family Hospital |
| ID698 | 8/5/2020 | ID698 | F0 | Asymptomatic | Danang Hospital; Lung Hospital; Ung Buou Hospital |
| ID699 | 8/5/2020 | ID699 | F0 | Asymptomatic | Danang Hospital |
| ID702 | 8/5/2020 | ID702 | F0 | Asymptomatic | Danang Hospital |
| ID718 | 8/6/2020 | ID718 | F0 | Asymptomatic | Danang Hospital; Family Hospital |
| ID737 | 8/6/2020 | ID737 | F0 | Asymptomatic | Danang Hospital |
| ID742 | 8/6/2020 | ID724 | F1 | Asymptomatic | None |
| ID758 | 8/7/2020 | ID758 | F0 | Asymptomatic | Danang Hospital |
| ID761 | 8/7/2020 | ID761 | F0 | Asymptomatic | Danang Hospital |
| ID764 | 8/7/2020 | ID764 | F0 | Asymptomatic | Danang Hospital |
| ID827 | 8/9/2020 | ID827 | F0 | Asymptomatic | Danang Hospital |
| ID957 | 8/16/2020 | ID957 | F0 | Asymptomatic | None |
| ID996 | 8/20/2020 | ID996 | F0 | Fever; Shortness of breath; Chills; Sore throat | Lung Hospital |
| ID1040 | 8/29/2020 | ID1040 | F0 | Shortness of breath | 199 Hospital; C Hospital |

**F0 was non-identifiable source COVID-19 infected patient, Fn the one who contracted the infection from Fn-1*

***Table S2*: Summary descriptive table by groups of level of contact tracing* *(Danang outbreak, July 2020)***

|  | **F0** | **F1** | **F2** | **F3** | **F4** | **p-value** |
| --- | --- | --- | --- | --- | --- | --- |
|  | ***N=220*** | ***N=104*** | ***N=40*** | ***N=22*** | ***N=3*** |  |
| **Gender** |  |  |  |  |  | 0.736 |
| Male | 91 (41.4%) | 39 (37.5%) | 17 (42.5%) | 6 (27.3%) | 1 (33.3%) |  |
| Female | 129 (58.6%) | 65 (62.5%) | 23 (57.5%) | 16 (72.7%) | 2 (66.7%) |  |
| **Age (year)** | 50.1 (17.2) | 44.0 (19.2) | 39.9 (18.4) | 45.3 (22.7) | 45.0 (15.7) | 0.004 |
| **Previously Covid-19 test** |  |  |  |  |  | 0.003 |
| Negative | 49 (22.3%) | 40 (38.5%) | 15 (37.5%) | 11 (50.0%) | 1 (33.3%) |  |
| Positive | 171 (77.7%) | 64 (61.5%) | 25 (62.5%) | 11 (50.0%) | 2 (66.7%) |  |
| **Quarantined before Covid-19 positive** |  |  |  |  |  | 0.014 |
| Yes | 106 (48.2%) | 67 (64.4%) | 25 (62.5%) | 16 (72.7%) | 2 (66.7%) |  |
| No | 114 (51.8%) | 37 (35.6%) | 15 (37.5%) | 6 (27.3%) | 1 (33.3%) |  |
| **Fever** |  |  |  |  |  | 0.407 |
| Yes | 22 (10.0%) | 13 (12.5%) | 5 (12.5%) | 1 (4.6%) | 0 (0.00%) |  |
| No | 198 (90.0%) | 91 (87.5%) | 35 (87.5%) | 21 (95.5%) | 3 (100%) |  |
| **Cough** |  |  |  |  |  | 0.764 |
| Yes | 10 (4.6%) | 4 (3.8%) | 0 (0.00%) | 0 (0.00%) | 0 (0.00%) |  |
| No | 210 (95.5%) | 100 (96.2%) | 40 (100%) | 22 (100%) | 3 (100%) |  |
| **Shortness of breath** |  |  |  |  |  | 0.556 |
| Yes | 2 (0.9%) | 1 (1.0%) | 0 (0.0%) | 0 (0.0%) | 0 (0.0%) |  |
| No | 218 (99.1%) | 103 (99.0%) | 40 (100%) | 22 (100%) | 3 (100%) |  |
| **Nasal congestion / runny nose** |  |  |  |  |  | 0.962 |
| Yes | 1 (0.5%) | 0 (0.0%) | 1 (2.5%) | 0 (0.0%) | 0 (0.0%) |  |
| No | 219 (99.5%) | 104 (100%) | 39 (97.5%) | 22 (100%) | 3 (100%) |  |
| **Myalgia** |  |  |  |  |  | 0.439 |
| Yes | 28 (12.7%) | 15 (14.4%) | 2 (5.0%) | 0 (0.0%) | 0 (0.0%) |  |
| No | 192 (87.3%) | 89 (85.6%) | 38 (95.0%) | 22 (100%) | 3 (100%) |  |
| **Chills** |  |  |  |  |  | 0.194 |
| Yes | 12 (5.5%) | 11 (10.6%) | 0 (0.0%) | 0 (0.0%) | 1 (33.3%) |  |
| No | 208 (94.5%) | 93 (89.4%) | 40 (100%) | 22 (100%) | 2 (66.7%) |  |
| **Sore throat** |  |  |  |  |  | 0.021 |
| Yes | 7 (3.2%) | 8 (7.7%) | 0 (0.0%) | 0 (0.0%) | 0 (0.0%) |  |
| No | 213 (96.8%) | 96 (92.3%) | 40 (100%) | 22 (100%) | 3 (100%) |  |
| **Headache** |  |  |  |  |  | 0.136 |
| Yes | 7 (3.2%) | 8 (7.7%) | 0 (0.0%) | 0 (0.0%) | 0 (0.0%) |  |
| No | 213 (96.8%) | 96 (92.3%) | 40 (100%) | 22 (100%) | 3 (100%) |  |
| **Taste_smell** |  |  |  |  |  | 0.627 |
| Yes | 1 (0.5%) | 2 (1.9%) | 0 (0.0%) | 0 (0.0%) | 0 (0.0%) |  |
| No | 219 (99.5%) | 102 (98.1%) | 40 (100%) | 22 (100%) | 3 (100%) |  |
| **Chest pain** |  |  |  |  |  | 0.004 |
| Yes | 5 (2.3%) | 1 (1.0%) | 2 (5.00%) | 0 (0.0%) | 1 (33.3%) |  |
| No | 215 (97.7%) | 103 (99.0%) | 38 (95.0%) | 22 (100%) | 2 (66.7%) |  |
| **Total symptoms** |  |  |  |  |  | 0.305 |
| 0 | 156 (70.9%) | 63 (60.6%) | 30 (75.0%) | 20 (90.9%) | 2 (66.7%) |  |
| 1 | 27 (12.3%) | 18 (17.3%) | 5 (12.5%) | 2 (9.1%) | 0 (0.0%) |  |
| 2 | 24 (10.9%) | 14 (13.5%) | 2 (5.0%) | 0 (0.0%) | 1 (33.3%) |  |
| 3 | 9 (4.1%) | 9 (8.7%) | 3 (7.5%) | 0 (0.0%) | 0 (0.0%) |  |
| 4 | 4 (1.8%) | 0 (0.0%) | 0 (0.0%) | 0 (0.0%) | 0 (0.0%) |  |
| **Common symptoms**** |  |  |  |  |  | 0.438 |
| 0 | 163 (74.1%) | 70 (67.3%) | 30 (75.0%) | 20 (90.9%) | 3 (100%) |  |
| 1 | 39 (17.7%) | 22 (21.2%) | 6 (15.0%) | 2 (9.09%) | 0 (0.0%) |  |
| 2 | 13 (5.9%) | 12 (11.5%) | 3 (7.5%) | 0 (0.0%) | 0 (0.0%) |  |
| 3 | 5 (2.3%) | 0 (0.0%) | 1 (2.5%) | 0 (0.0%) | 0 (0.0%) |  |
| **Less common symptoms***** |  |  |  |  |  | 0.073 |
| 0 | 201 (91.4%) | 86 (82.7%) | 39 (97.5%) | 22 (100%) | 2 (66.7%) |  |
| 1 | 17 (7.7%) | 15 (14.4%) | 1 (2.5%) | 0 (0.0%) | 1 (33.3%) |  |
| 2 | 2 (0.9%) | 3 (2.9%) | 0 (0.0%) | 0 (0.0%) | 0 (0.0%) |  |
| **Severe symptoms^⁂^** |  |  |  |  |  | 0.387 |
| 0 | 206 (93.6%) | 100 (96.2%) | 38 (95.0%) | 22 (100%) | 2 (66.7%) |  |
| 1 | 13 (5.91%) | 3 (2.9%) | 2 (5.0%) | 0 (0.0%) | 1 (33.3%) |  |
| 2 | 1 (0.45%) | 1 (1.0%) | 0 (0.0%) | 0 (0.0%) | 0 (0.0%) |  |

*Statistical analysis test: a: Fisher’s exact test, b: Phi and Cramer’s V.*

*Descriptive information was reported as a: N (%), b: mean (SD)*

**F0 was non-identifiable source COVID-19 infected patient, Fn the one who contracted the infection from Fn-1*

***Common symptoms: fever, cough, chills. ***Less common symptoms: myalgia, sore throat, diarrhea, red eyes, headache, loss of taste or smell, rash or redness, or cyanosis of the fingers or toes.* **^⁂^** *Severe symptoms: needing the intensive care unit (ICU) or mechanical ventilation, severe dyspnea up to inability of taking breath, persistent chest pain or pressure, hypoxia or hypoxemia up to the loss of consciousness level of confusion, loss of ability to move or talk in addition to cardiac arrest, severe arrhythmia, neural epilepsy or seizures.*

**Table S3: Summary of Uni- and multivariable logistic regression analysis for death patients (Danang outbreak, July 2020).**

|  | Univariate | | Multivariable regression | | |  |
| --- | --- | --- | --- | --- | --- | --- |
|  |  |  | **Model 1** | | **Model 2** |  |
|  | **OR (95% CI)** | **p-value** | **OR (95% CI)** | **p-value** | **OR (95% CI)** | **p-value** |
| (Intercept) | - | - | 0.00 (0.00 – 0.05) | **<0.001** | 0.01 (0.00 – 0.03) | **<0.001** |
| Age | 1.76 (1.40 – 2.26) | **< 0.001** | 1.78 (1.39 – 2.36) | **<0.001** | 1.83 (1.43 – 2.41) | **<0.001** |
| Previously negative test |  |  |  |  |  |  |
| No | *Reference* | | *Reference* | | *Reference* |  |
| Yes | 0.06 (0.00 – 0.30) | **0.007** | 0.07 (0.00 – 0.34) | **0.009** | 0.06 (0.00 – 0.29) | **0.006** |
| Quarantined before COVID-19 positive |  |  |  |  |  |  |
| No | *Reference* | | *Reference* | | *Reference* |  |
| Yes | 0.30 (0.13-0.63) | **0.002** | 0.34 (0.14 – 0.79) | **0.015** | 0.32 (0.13 – 0.72) | **0.008** |
| Severe Symptoms* |  |  |  |  |  |  |
| No | *Reference* | | *Reference* | | *Reference* |  |
| Yes | 6.31 (2.23 – 16.59) | **<0.001** | 4.81 (1.33 – 17.18) | **0.015** | 5.09 (1.45 – 17.66) | **0.010** |
| Source of infection** |  |  |  |  |  |  |
| Undefined (F0) | *Reference* | | *Reference* | | *Reference* |  |
| Defined (F1, F2, F3, F4) | 0.20 (0.10 – 0.22) | **0.001** | 0.38 (0.12 – 1.01) | 0.069 | - | - |

******Severe symptoms: needing the intensive care unit (ICU) or mechanical ventilation, severe dyspnea up to inability of taking breath, persistent chest pain or pressure, hypoxia or hypoxemia up to the loss of consciousness level of confusion, loss of ability to move, or talk in addition to cardiac arrest, severe arrhythmia, neural epilepsy or seizures.*

*** F0 was non-identifiable source COVID-19 infected patient, Fn the one who contracted the infection from Fn-1*
